# A novel machine learning strategy for model selections - Stepwise Support Vector Machine (StepSVM)

**DOI:** 10.1371/journal.pone.0238384

**Published:** 2020-08-27

**Authors:** Chao-Yu Guo, Yu-Chin Chou

**Affiliations:** Institute of Public Health, School of Medicine, National Yang-Ming University, Taipei, Taiwan; University of Craiova, ROMANIA

## Abstract

An essential aspect of medical research is the prediction for a health outcome and the scientific identification of important factors. As a result, numerous methods were developed for model selections in recent years. In the era of big data, machine learning has been broadly adopted for data analysis. In particular, the Support Vector Machine (SVM) has an excellent performance in classifications and predictions with the high-dimensional data. In this research, a novel model selection strategy is carried out, named as the Stepwise Support Vector Machine (StepSVM). The new strategy is based on the SVM to conduct a modified stepwise selection, where the tuning parameter could be determined by 10-fold cross-validation that minimizes the mean squared error. Two popular methods, the conventional stepwise logistic regression model and the SVM Recursive Feature Elimination (SVM-RFE), were compared to the StepSVM. The Stability and accuracy of the three strategies were evaluated by simulation studies with a complex hierarchical structure. Up to five variables were selected to predict the dichotomous cancer remission of a lung cancer patient. Regarding the stepwise logistic regression, the mean of the C-statistic was 69.19%. The overall accuracy of the SVM-RFE was estimated at 70.62%. In contrast, the StepSVM provided the highest prediction accuracy of 80.57%. Although the StepSVM is more time consuming, it is more consistent and outperforms the other two methods.

## Introduction

There are two types of machine learning, the supervised machine learning with a specific outcome variable and the unsupervised machine learning that only examines the associations between a set of predictors [1]. Regression and classification are two primary applications for supervised learning, such as the generalized linear model (GLM) [2], the logistic regression model [3], and the Support Vector Machine (SVM) [4]. For unsupervised learning, clustering is the leading interest and the most popular method is the Principal Components Analysis (PCA) [5].

The SVM is a machine learning tool dealing with classification problems. With an increasing amount of variables collected, the high dimensional data draw more attention in image processing and the SVM is considered a powerful classification method. Chang et al. concluded that the SVM is useful in the imaging diagnosis of breast cancer and its classification ability is nearly equal to a neural network model [6]. In particular, when a non-linear structure exists, the SVM demonstrates its superior ability to find the optimal separating hyperplane by kernel tricks into a higher dimensional feature space [7].

One powerful application of the SVM is the model selection. However, the conventional logistic regression using the concordance statistic (C-statistic or C-index) [8] is capable of various types of model selection based on the Mann-Whitney U statistic [9]. The machine learning technique is somewhat desired [10] since the kernel function is powerful in classification problems [11]. In 2002, a new methodology addressed the problem of the selection of a small subset of genes from broad patterns of gene expression data, recorded on DNA microarrays [12]. Using available training examples from cancer and healthy patients, they built a classifier suitable for genetic diagnosis, as well as drug discovery. In contrast to previous attempts that addressed this problem of selecting genes with the correlated structure, they carried out a new method of gene selection utilizing the Support Vector Machine based on Recursive Feature Elimination (SVM-RFE) by re-weighting the genes using backward eliminations.

The prediction performances of the SVM based on different kernel functions were compared by Huang et al. [13]. This study suggests that the linear kernel-based SVM ensembles based on the bagging method and the RBF kernel-based SVM ensembles based on the boosting method could be the better choices for a small scale dataset if feature selection is performed in the pre-processing data stage. For a large scale dataset, the RBF kernel-based SVM ensembles based on the boosting method perform better than the other classifiers.

The SVM is also a popular winner in genetic studies. Zhi et al. [14] selected candidate genes by the SVM classifier based on the betweenness centrality (BC) algorithm. Colorectal cancer (CRC) dataset from the Cancer Genome Atlas database was used to evaluate the accuracy of the SVM classifier. Pathway enrichment analysis was carried out for the SVM-classified gene signatures.

Recently, Battineni et al. [15] applied the SVM on MRI (Magnetic Resonance Image) data to predict dementia patients. Through deliberate statistical analyses, they pointed out the importance of parameter tuning in the SVM. Their results showed substantial evidence that better performance values for dementia prediction could be accomplished by low gamma (1.0E-4) and high regularized (C = 100) values. Undoubtedly, applications of the SVM are quite extensive in various research fields.

Although the SVM has been widely extended in a variety of concepts, such as the parameter tuning or kernel choices, this research is focusing on statistical methodologies for stepwise selection models. The backward eliminations implemented by the SVM-RFE consider variables that are top-ranked to be eliminated last are not necessarily the factors that are individually most relevant. In particular, these predictors are the most relevant conditional on the specific ranked subset in the model. In order to avoid incorrectly eliminated factors, we propose a novel forward algorithm with stepwise considerations based on the SVM. The name of the new strategy is the Stepwise Support Vector Machine (StepSVM).

The performances of each method could be evaluated by accuracy ($Accuracy=\frac{Ture\;Positive+Ture\;Negative}{Total\;Number\;Of\;Tests}$). Cross-validation (CV) will be implemented to assess accuracy [16]. In particular, the 10-fold CV is the one we adopted according to the previous study [17].

## Materials and methods

The SVM classifies subjects according to the separating hyperplane, which is defined as ***ω***^*T*^***x***_*i*_+*b* = 0, where ***ω***^*T*^***x***_*i*_+*b*≥1,∀*y*_*i*_ = 1 and ***ω***^*T*^***x***_*i*_+*b*≤−1,∀*y*_*i*_ = −1. It could be rewritten as *y*_*i*_(***ω***^*T*^***x***_*i*_+*b*)≥1, *i* = 1⋯*m*, with the $\mathrm{Maximum}\;\mathrm{Margin}=\underset{\omega ,b}{\max}\frac{2}{\left\|\omega \right\|}$ or $\underset{\omega ,b}{\min}\frac{1}{2}\|\omega \|$.

With a restriction C (Cost), *y*_*i*_(***ω***^*T*^***x***_*i*_+*b*)≥1−*ξ*_*i*_, *ξ*_*i*_≥0,*i* = 1⋯*m*, with the maximum margin becomes $\underset{\omega ,b}{\min}\frac{1}{2}\|\omega \|+C\sum_{i=1}^{m}\xi_{i}$. In order to obtain the maximum value, the Lagrange Multiplier Method is applied, Lagrangiansis $L(\omega ,b,\xi ,\alpha ,\beta )=\frac{1}{2}{\left\|\omega \right\|}^{2}+C\sum_{i=1}^{m}\xi_{i}-\sum_{i=1}^{m}\alpha_{i}\times [{y_{i}(\omega }^{T}x_{i}+b)+1-\xi_{i}]-\sum_{i=1}^{m}{\beta_{i}\xi }_{i}$, where *α*_*i*_ and *β*_*i*_ are the Lagrange Multipliers.

According to Karush-Kuhn and Tucker (KKT), partial derivatives of Lagrangian with respect to ***ω***, *b*, and *ξ* equals zero.

1. $\frac{\partial }{\partial \omega }L(\omega ,b,\xi ,\alpha ,\beta )=0$
  $$\frac{\partial }{\partial \omega }\frac{1}{2}\omega^{T}\omega +C\sum_{i=1}^{m}\xi_{i}-\sum_{i=1}^{m}\alpha_{i}\times [y_{i}(\omega^{T}x_{i}+b)+1-\xi_{i}]-\sum_{i=1}^{m}\beta_{i}\xi_{i}=0$$
  $$\omega -\sum_{i=1}^{m}\alpha_{i}y_{i}x_{i}=0$$
  $$\omega =\sum_{i=1}^{m}\alpha_{i}y_{i}x_{i}$$
2. $\frac{\partial }{\partial b}L(\omega ,b,\xi ,\alpha ,\beta )=0$
  $$\frac{\partial }{\partial b}\frac{1}{2}\omega^{T}\omega +C\sum_{i=1}^{m}\xi_{i}-\sum_{i=1}^{m}\alpha_{i}\times [y_{i}(\omega^{T}x_{i}+b)+1-\xi_{i}]-\sum_{i=1}^{m}\beta_{i}\xi_{i}=0$$
  $$\sum_{i=1}^{m}\alpha_{i}y_{i}=0$$
3. $\frac{\partial }{\partial \xi }L(\omega ,b,\xi ,\alpha ,\beta )=0$
  $$\frac{\partial }{\partial \xi }\frac{1}{2}\omega^{T}\omega +C\sum_{i=1}^{m}\xi_{i}-\sum_{i=1}^{m}\alpha_{i}\times [y_{i}(\omega^{T}x_{i}+b)+1-\xi_{i}]-\sum_{i=1}^{m}\beta_{i}\xi_{i}=0$$
  $$C-\alpha_{i}-\beta_{i}=0$$
  $$\mathrm{Finally},\;\Theta \left(\alpha ,\beta \right)=-\frac{1}{2}\sum_{i=1}^{m}\sum_{j=1}^{m}\alpha_{i}\alpha_{j}y_{i}y_{j}x_{i}^{T}x_{j}+\sum_{i=1}^{m}\alpha_{i}$$
  $$\underset{\alpha }{\max}\sum_{i=1}^{m}\alpha_{i}-\frac{1}{2}\sum_{i=1}^{m}\sum_{j=1}^{m}\alpha_{i}\alpha_{j}y_{i}y_{j}x_{i}^{T}x_{j}$$
  $$subject\;to\sum_{i=1}^{m}\alpha_{i}y_{i}=0,\;0\leq \alpha_{i}\leq C$$

If the raw data are not linearly separable, the kernel function solves the classification problem in a higher-dimensional space or an infinite-dimensional space, such as the RBF kernel [18]. The kernel function is defined as *K*(**x**,**x**′) = *ϕ*(**x**)^*T*^*ϕ*(**x**′). For the linear kernel, *K*(**x**,**x**′) = 〈**x**,**x**′〉. Regarding the non-linear one, Radial Basis Function (RBF) kernel *K*(**x**,**x**′) = exp (−*γ*‖**x**−**x**′‖^2^) is commonly adopted. *γ* is greater than zero and the choice has been discussed previously [19]. Finally, the optimal separating hyperplane is given by $\omega^{T}x_{i}+b={\left(\sum_{i=1}^{m}\alpha_{i}y_{i}x_{i}\right)}^{T}x_{i}+b=\sum_{i=1}^{m}\alpha_{i}y_{i}{<x}_{i},x_{i}>+b{=\sum_{i=1}^{m}\alpha_{i}y_{i}\phi (x_{i})}^{T}\phi (x_{i})+b=\sum_{i=1}^{m}\alpha_{i}y_{i}K(x_{i},x_{i})+b$.

Two popular model selection strategies are compared to the StepSVM. The first one is the conventional logistic regression with stepwise selection since it is considered the gold standard for classification problems. The other one is the backward elimination method, the SVM-RFE. Note that the logistic regression model uses the aggregate data, the same as the traditional statistical approach. However, the two SVM methods separate the original data into 80% training and 20% testing data with a 10-fold CV.

The simulation study is based on a hierarchical dataset using the R code from the University of California, Los Angeles Institute for Digital Research and Education (https://stats.idre.ucla.edu/r/codefragments/mesimulation/), where patients are nested in doctors, and doctors are nested in hospitals. The number of hospitals (HID) is 35, and each hospital has 8 to 15 doctors (DID). Finally, 2 to 40 patients (ID) are randomly generated for each doctor.

As a result, the expected sample size is 8525 with 26 predictors, and the dichotomous remission variable is the primary outcome, and the hierarchical structure is listed in A1 Table in S1 Appendix. Details of all variables are displayed in A2 Table in S1 Appendix. Descriptive statistics for continuous variables are in A3 Table in S1 Appendix. Categorical variables are described in A4 Table in S1 Appendix (except the I.D. variables, DID and HID). One hundred repetitions were conducted to evaluate the performances of the three methods. Note that the sample size varies slightly due to random assignments of the number of doctors and patients.

In clinical research, the prediction model usually prefers a simple scoring system with 5 to 10 significant factors. In our previous work, we implemented the SVM as the primary statistical model with only six predictors to examine the clinical prediction ability in an independent replication study [20]. Therefore, in computer simulations, up to 5 variables were selected from a total of 26 variables to compare the three strategies. Results are displayed as the order being selected by the model, where the coding numbers are in A5 Table in S1 Appendix.

The conventional stepwise selection was used by the logistic regression model, which did not require parameter tuning. For the SVM-RFE and StepSVM, both linear and RBF kernels with various combinations of C and γ were evaluated by 10-fold CV. However, only those parameters with the best performance were chosen for comparisons in the simulation study. Hence, the SVM-RFE adopted the Linear kernel and the optimal C value was 0.1 according to the tune(.) function. The StepSVM was also based on the 10-fold CV for parameter tuning and the RBF Kernel was selected with C = 10 and γ = 1. A sensitivity analysis for the SVM-RFE could ensure fair comparisons between the SVM-RFE and StepSVM. Therefore, an additional setting with C = 10 was also examined for the SVM-RFE.

The StepSVM is intuitive and consists of only a few simple steps. In A1 Fig in S1 Appendix, the flow chart of the algorithm implemented for the StepSVM is presented. The first procedure of the StepSVM examines all possible combinations of any two variables from the 26 (m) variables, and the total possibilities are $C_{2}^{m}=\frac{m!}{(m-2)!}$. The combination with the highest accuracy is selected as the first two components of the StepSVM. Next, in the remaining (m−2) = 26−2 = 24 variables, sequentially add one variable to the first two components. Among the 24 scenarios, the one with the best accuracy is selected as the third component. The following iterations repeat the previous step, which aims to add in the next most influential factor after those being selected from the previous step. This procedure of selecting the next component repeats until the accuracy no longer increases, or the maximum number of components allowed is reached. In this research, up to 5 variables are allowed, since there are only 26 predictors that could be evaluated. In summary, the first procedure consists of 325 possibilities. The 2^nd^ step reduced to 24 models, and the 3^rd^ step further goes down to 23 variables. Finally, the 5^th^ variables are selected from 22 possibilities.

## Results

**Fig 1:**
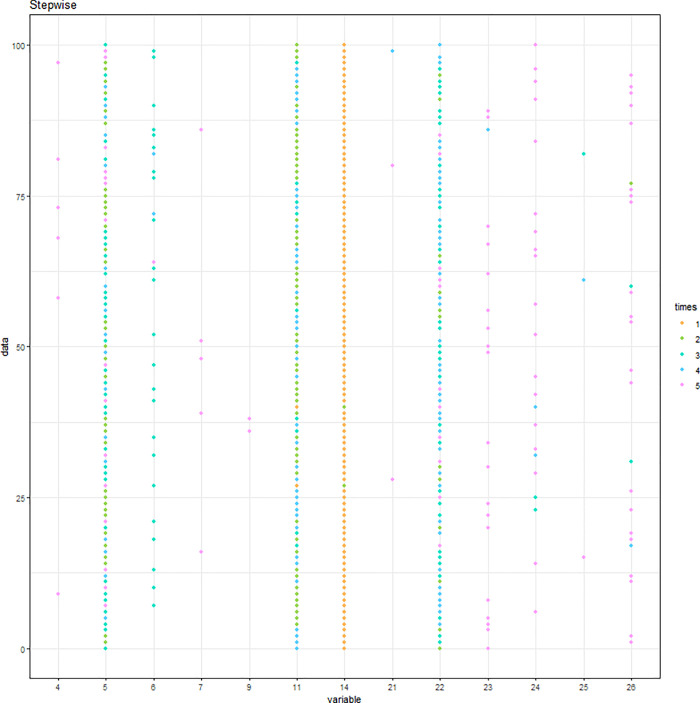
Variables being selected by the stepwise logistic regression model.

**Fig 2:**
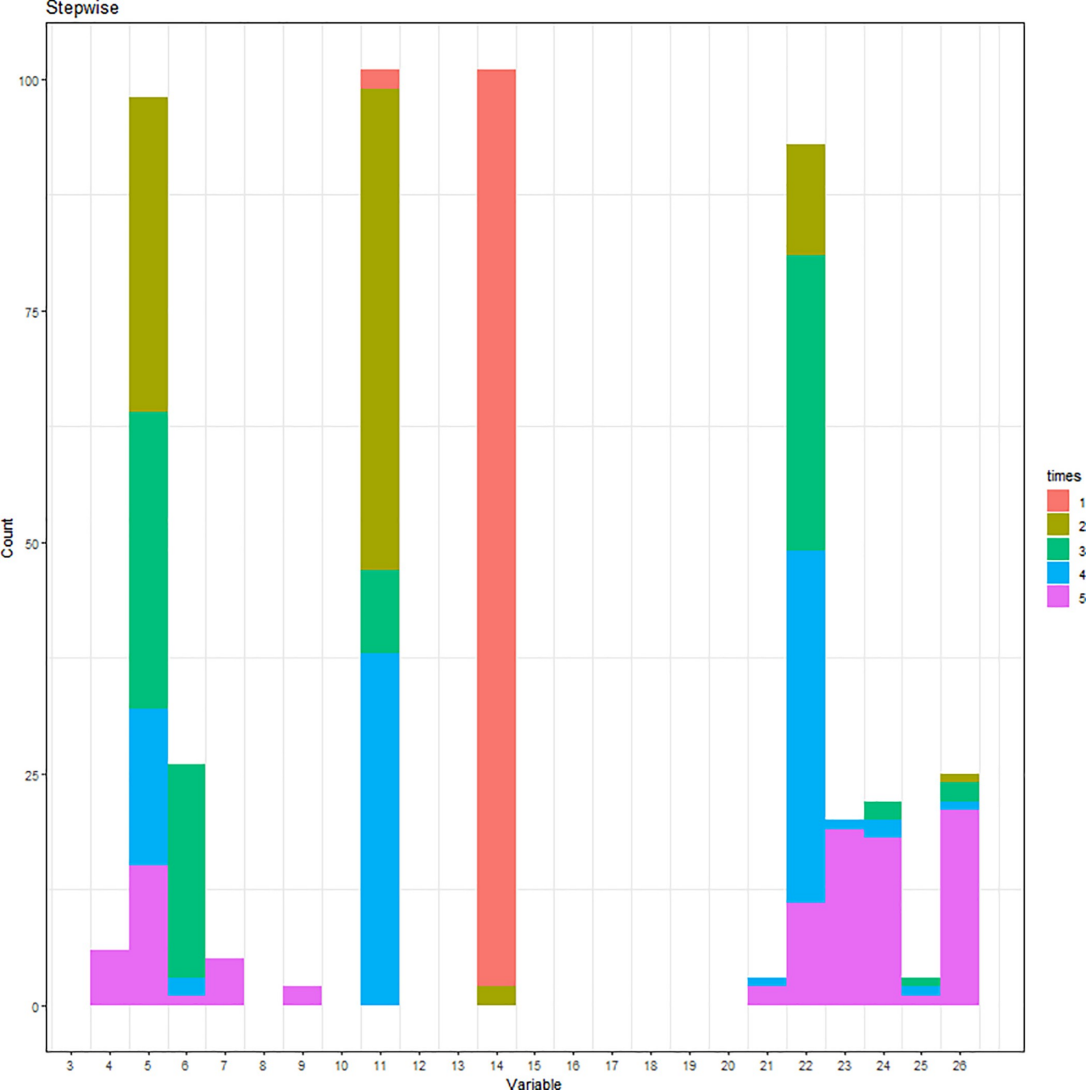
Variables being selected by the stepwise logistic regression model (cumulative frequency).

**Fig 3:**
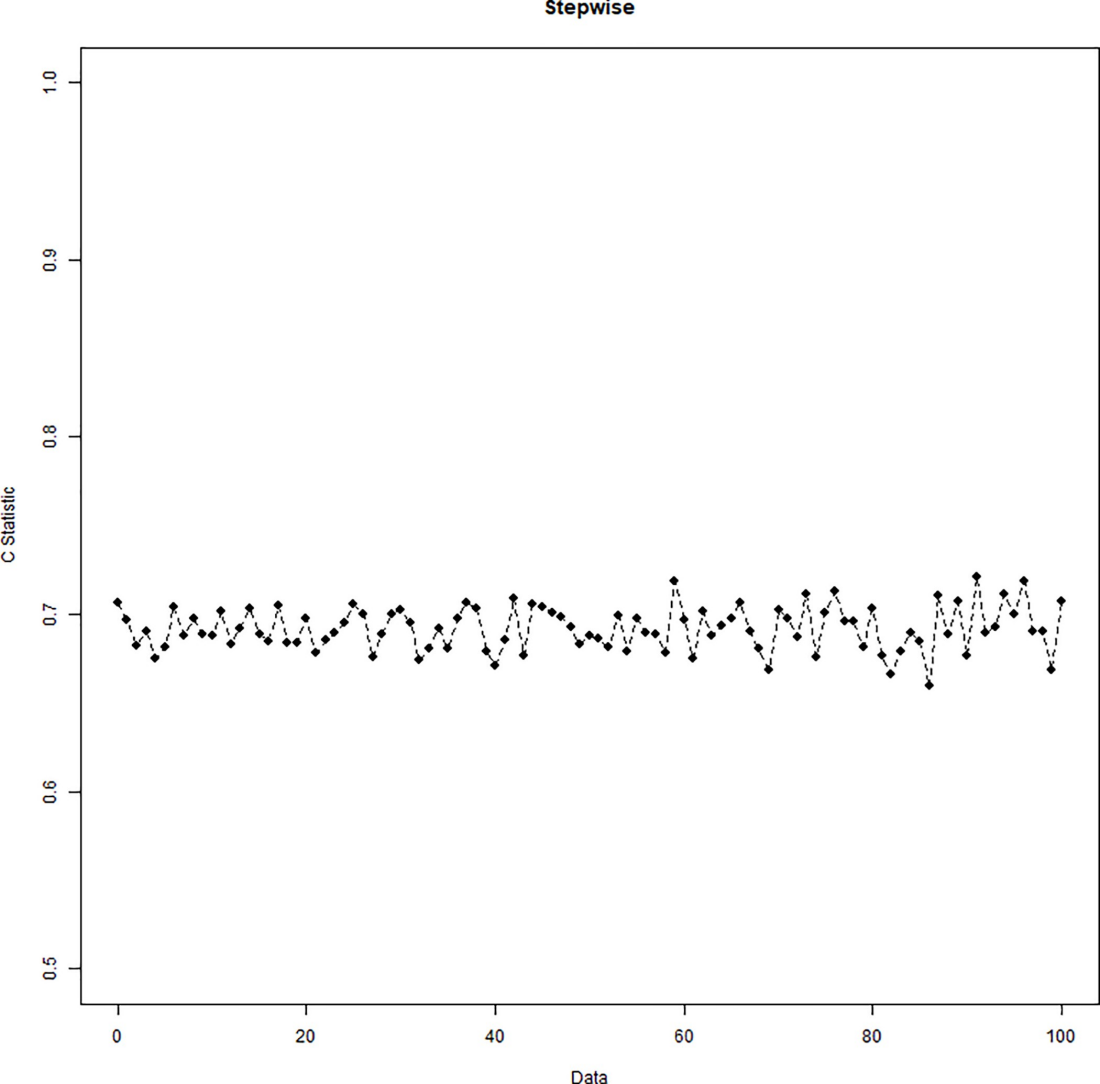
C-statistic of the stepwise logistic regression model.

Simulation results of the stepwise logistic regression model are displayed in Fig 1, where different colors note the order of five variables being selected among the 100 repetitions. Variables with the highest values of frequency are mobility, FamilyHx, CancerStage, and Experience. The first variable being selected with the highest frequency is CancerStage, followed by mobility, FamilyHx, and then Experience. However, the 5^th^ variable varies and is not consistently chosen. The cumulative frequency of variables being selected is displayed in Fig 2. The C-statistic of the 100 repetitions is displayed in Fig 3.

**Fig 4:**
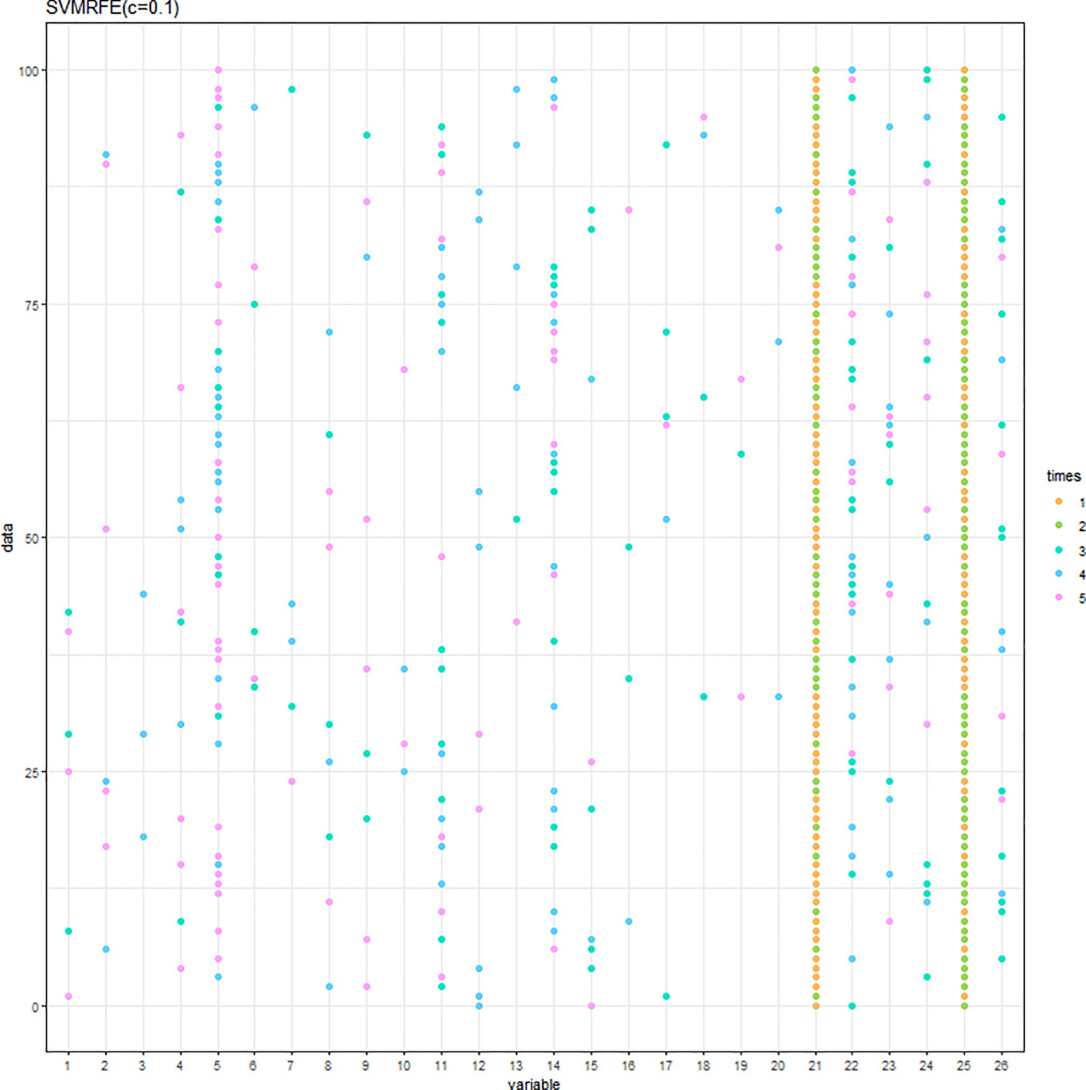
Variables being selected by the SVM-RFE (C = 0.1).

**Fig 5:**
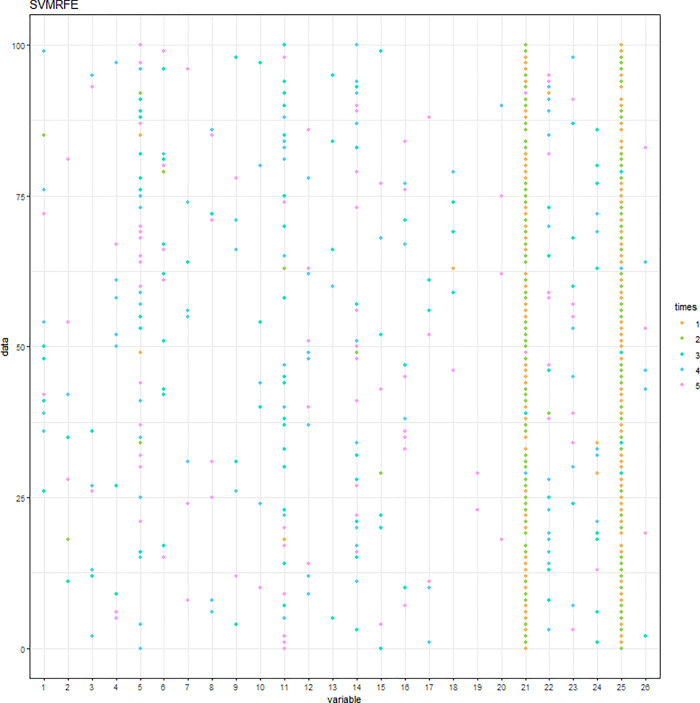
Variables being selected by the SVM-RFE (cumulative frequency) (C = 0.1).

**Fig 6:**
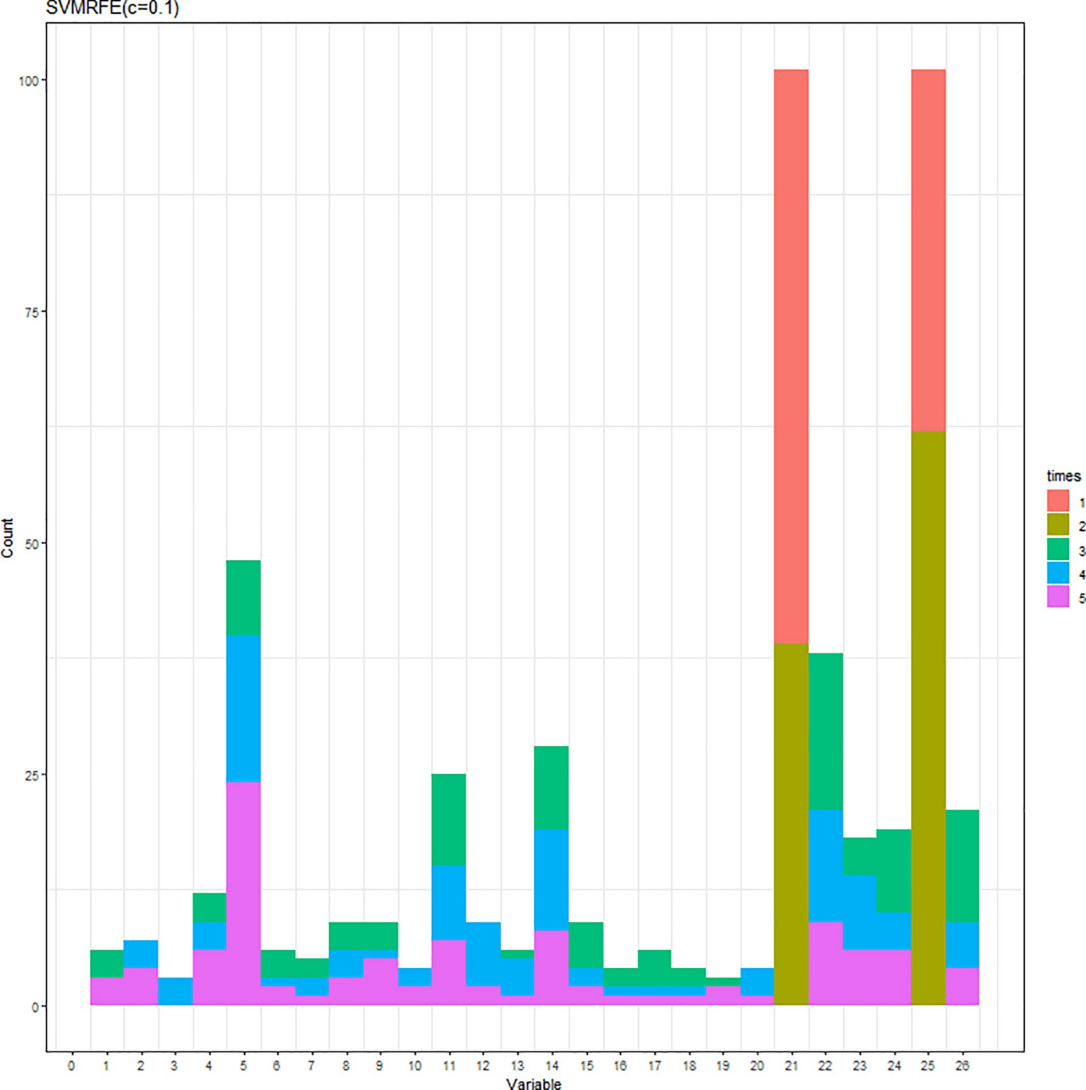
Accuracy of the SVM-RFE (C = 0.1).

**Fig 7:**
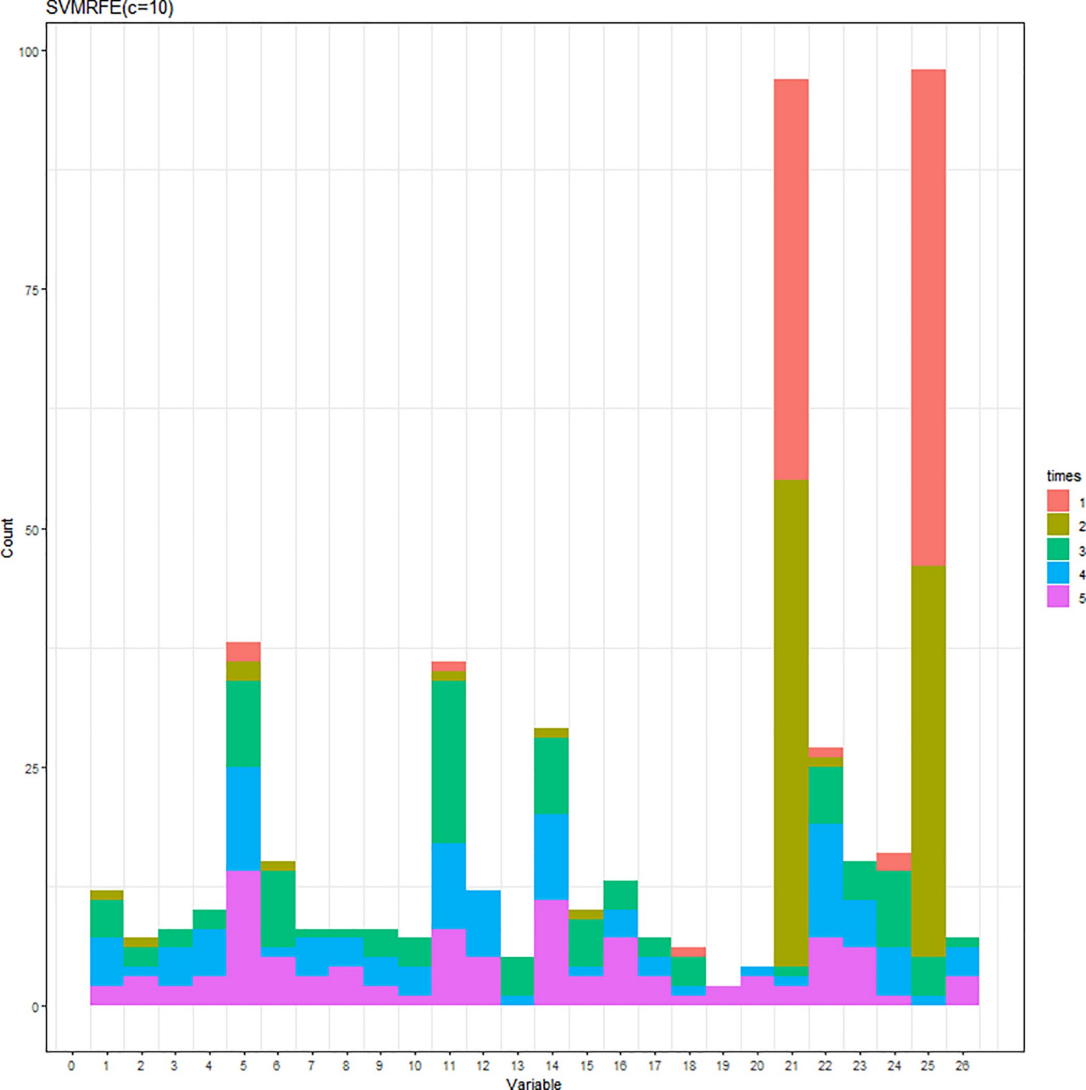
Variables being selected by the SVM-RFE (C = 10).

**Fig 8:**
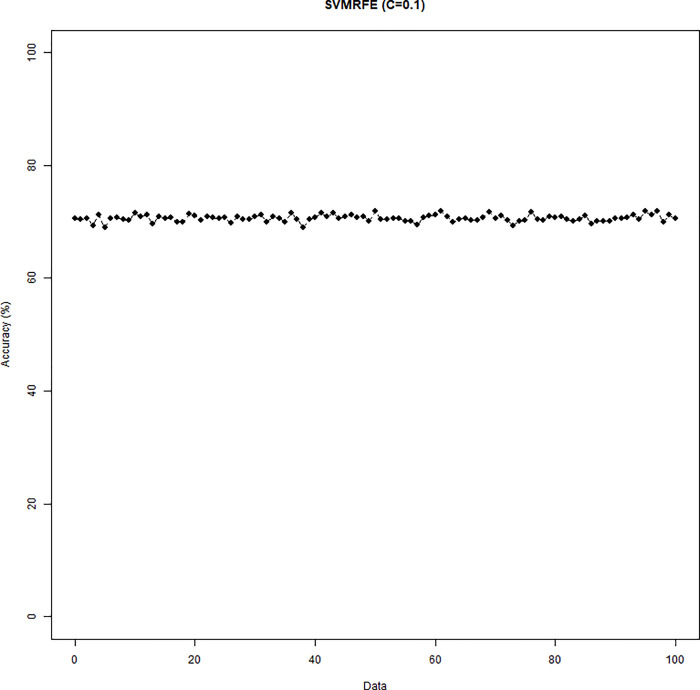
Accuracy of the SVM-RFE (C = 0.1).

**Fig 9:**
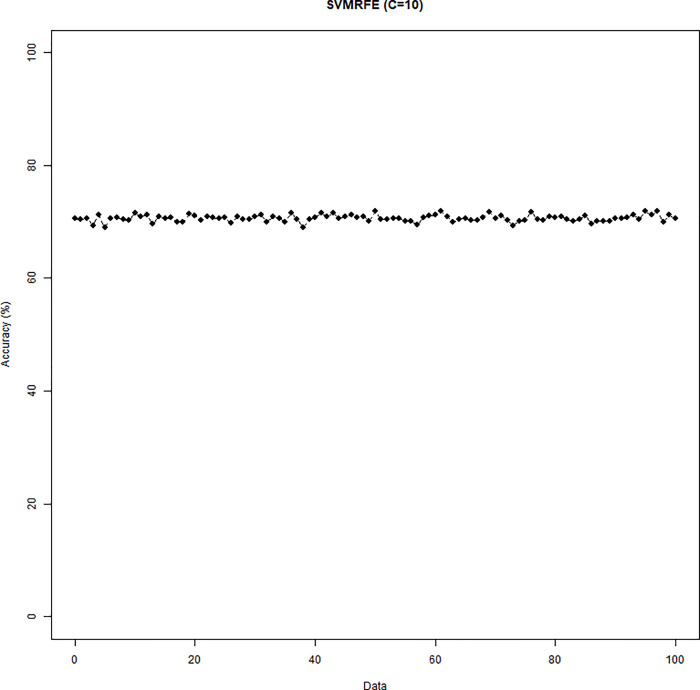
Accuracy of the SVM-RFE (C = 10).

The SVM-RFE with C = 0.1 or C = 10 was conducted. When C = 0.1 (C = 10), the results of the variable selection are displayed in Fig 4 (Fig 5). Regardless of the C parameter, DID and HID are generally the first two variables being selected and the 3^rd^ to the 5^th^ variables are not consistent. The cumulative frequency of variables being selected is displayed in Fig 6 (Fig 7). When C = 0.1 (C = 10), the accuracies of the 100 repetitions are displayed in Fig 8 (Fig 9). Therefore, different values of the C parameter yielded similar results, but the set of 5 variables was consistent, and the accuracy was unchanged.

**Fig 10:**
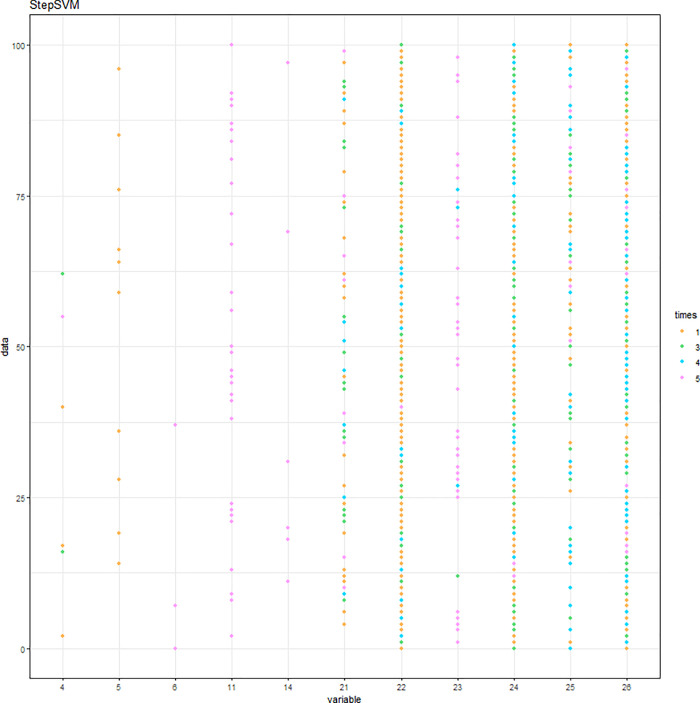
Variables being selected by the StepSVM.

**Fig 11:**
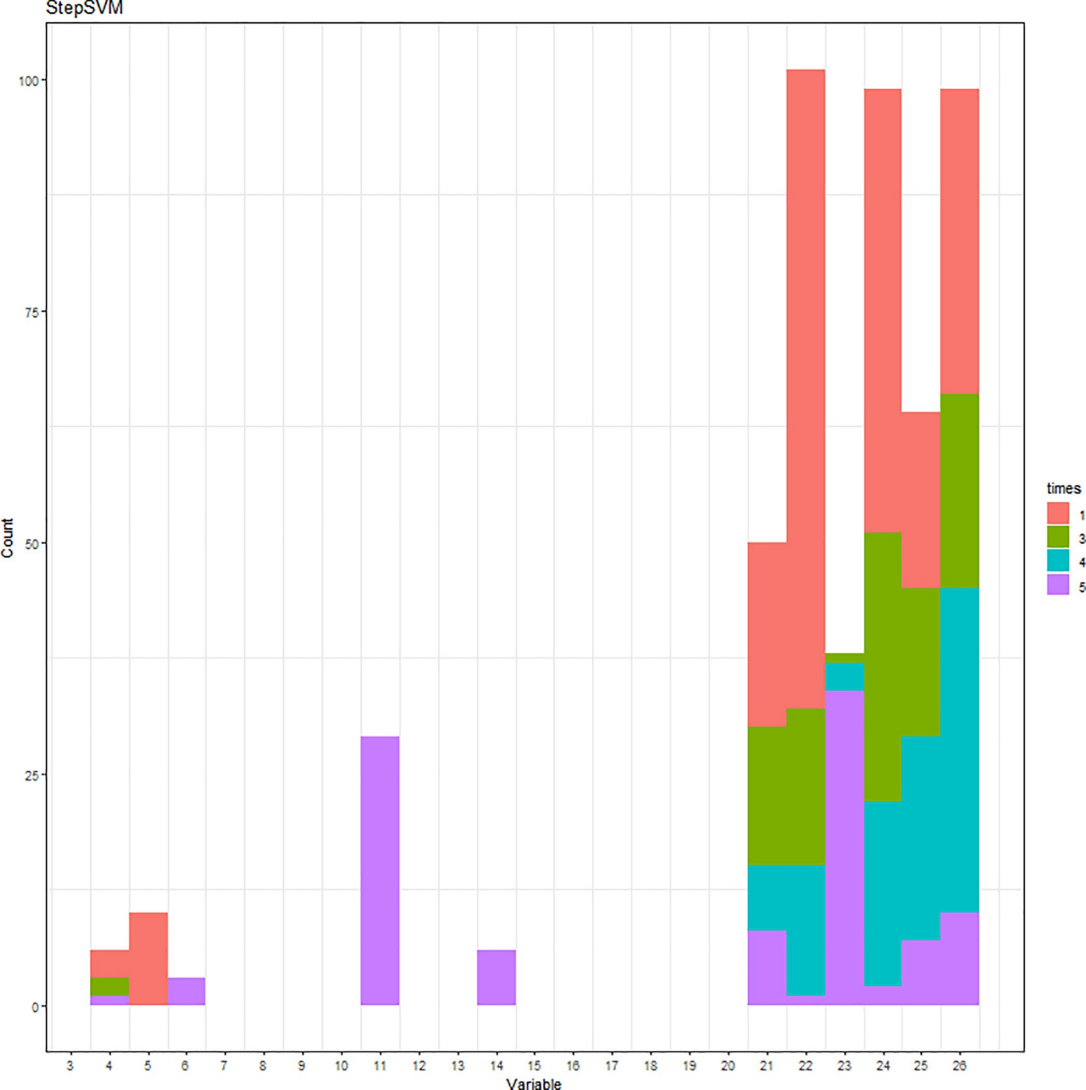
Variables being selected by the StepSVM (cumulative frequency).

**Fig 12:**
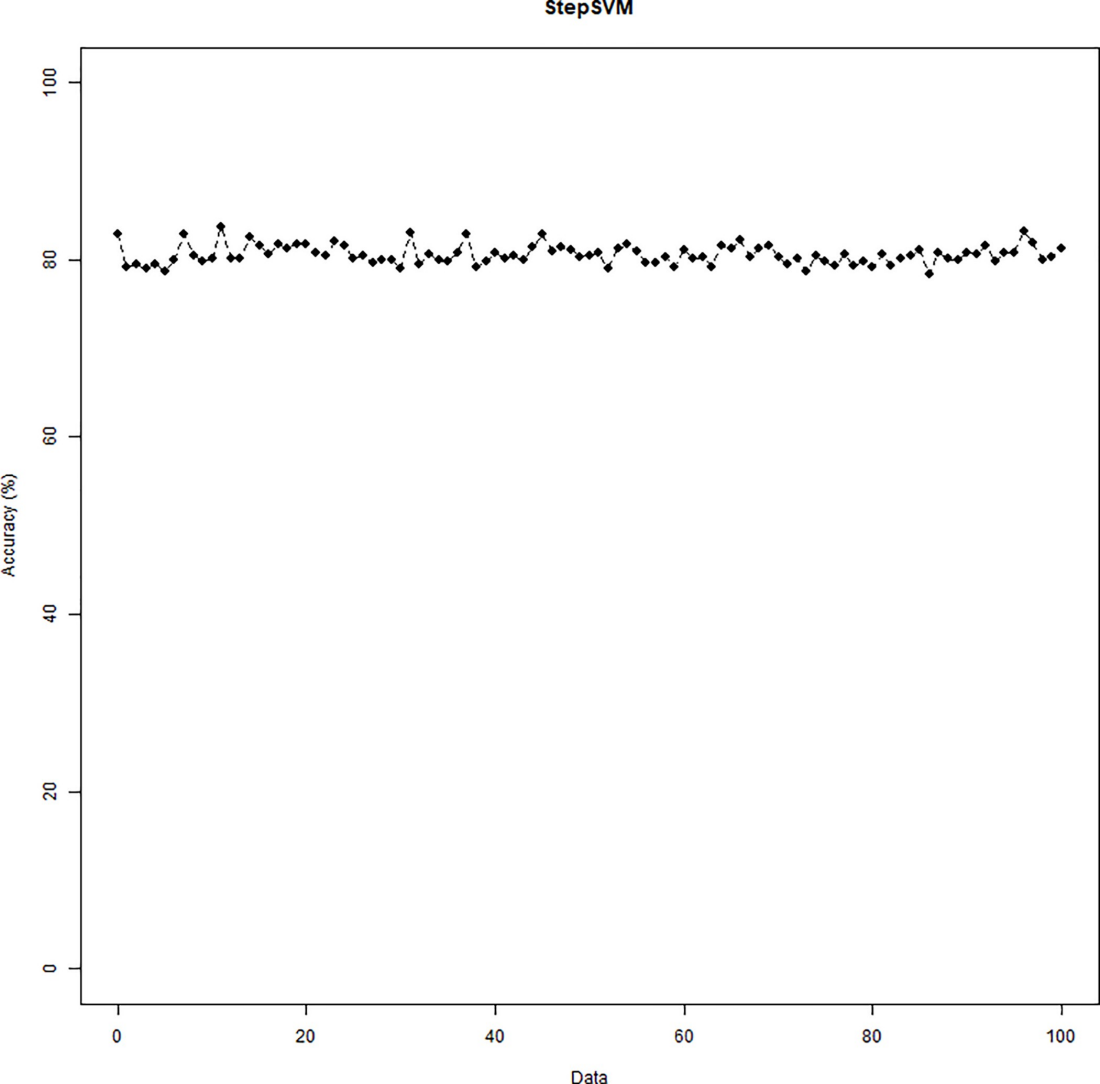
Accuracy of the StepSVM.

The simulation results of the StepSVM are displayed in Fig 10. Note that the combination of two variables is the first step being selected by the StepSVM. Unlike the other two methods, the 3^rd^ to the 5^th^ variables being selected were entirely consistent (DID, Experience, and Medicaid). Cumulative frequencies are displayed in Fig 11. Accuracies of the 100 repetitions are shown in Fig 12.

In summary, the top five variables being selected by three different methods with the highest frequency are listed in Table 1. The first variable selected by each method is highly associated with the outcome. CancerStage, DID, and Experience are picked by the logistic regression, the SVM-RFE, and the StepSVM, respectively.

**Table 1:** Variables being selected with the highest frequency.

Method	Parameter setting	Variable
Stepwise		CancerStage, Experience, mobility, FamilyHx, School
SVM-RFE^a^	C = 0.1	DID, HID, Experience, SmokingHx, LengthofStay
	C = 10	DID, HID, LengthofStay, mobility, FamilyHx
StepSVM^b^	C = 10, γ = 1	Experience, Medicaid, Lawsuits, HID, ntumors

^a^Using Linear kernel

^b^Using Radial basis function kernel

The performance of logistic regression is assessed by the C-Statistic (Table 2), while the SVM-RFE and StepSVM are evaluated by accuracy. With the restriction of up to five variables being selected, the SVM-RFE came up with the same set of five variables that generated the same accuracy, regardless of the regularization parameter C. The logistic regression yielded the average C-statistic 70.69%. In contrast, the average accuracy for the SVM-RFE and StepSVM is 70.65% and 80.12%, respectively. It is worth noting that the StepSVM provided the best accuracy.

**Table 2:** Performance of the three methods.

Method	Parameter setting	C-statistic / Accuracy
Stepwise		C-statistic = 70.69%
SVM-RFE^a^	C = 0.1	Accuracy = 70.65%
	C = 10	Accuracy = 70.65%
StepSVM^b^	C = 10, γ = 1	Accuracy = 80.12%

^a^Using Linear kernel

^b^Using Radial basis function kernel

## Discussions

Machine learnings are gaining popularity with astonishing speed in all kinds of research. In this study, a novel methodology for the forward stepwise model selection based on the famous SVM is carried out. Unlike the backward elimination scheme, which may erroneously remove variables based on conditional relevance, the StepSVM is intuitive with only a few simple stages and thus could be implemented easily.

According to simulations studies, even with a complicated hierarchical structure, the StepSVM provided the highest accuracy, and the variables being selected were much more consistent. This new method may contribute significantly to various research fields such as medicine, clinical research, public health, or environmental health when the selection of a handful of predictors is desired to create an optimal prediction model.

The only cost of the StepSVM is the computer execution time since it requires $C_{2}^{m}+\frac{1}{2}(m-2)\times (m-1)$ times of the SVM analyses. In contrast, the stepwise logistic regression has only $\frac{1}{2}m\times (m+1)$ choices. The SVM-RFE has the least (m) times, and it is the fastest procedure.

It is worth noting that the simulation studies are quite time-consuming. Although only 100 repetitions were conducted, the results and conclusions were very consistent. Further repetitions do not alter the conclusions. Besides, the StepSVM determines the most important factor based on the highest accuracy. However, if the sensitivity requires more attention than the other, one could easily alter the selection scheme with the highest sensitivity. Similarly, the highest specificity could also be adopted in the selection procedure. Hence, the StepSVM is not only simple but also very flexible and could be easily extended for more methodological researches.

To date, the SVM is extensively implemented by a variety of concepts in order to accomplish many more complex problems. Since this research focuses on statistical methodologies with a stepwise selection technique, comparisons are limited to the statistical methods related to this topic. Therefore, future studies are needed to examine further the application of the StepSVM in various research fields such as the genome-wide association studies (GWAS) or a high dimensional big data.

## Supporting information

S1 Appendix (DOCX)
