# Supplementary material for: A novel machine learning strategy for model selections - Stepwise Support Vector Machine (StepSVM)
Source: PLoS One. 2020 Aug 27;15(8):e0238384. doi: 10.1371/journal.pone.0238384 (PMC7451646; doi:10.1371/journal.pone.0238384)
Supplement: S1 Appendix — (DOCX) [file pone.0238384.s001.docx]

APPENDIX

#### **Table A1. The hierarchical structure of predictors associated with cancer remission**

| **Outcome variable** | **Level 1 Predictors** | **Level 2 Predictors** | **Level 3 Predictors** |
| --- | --- | --- | --- |
| Cancer remission | - Age | - DID | - HID |
|  | - LengthofStay | - Experience |  |
|  | (random by DID) |  |  |
|  | - FamilyHx |  |  |
|  | - IL6 |  |  |
|  | - CRP |  |  |
|  | - CancerStage |  |  |

**Table A2: Definitions of variables**

| **Variables** | **Data type** | **Definitions** |
| --- | --- | --- |
| remission | Categorical | Cancer in remission (TRUE/FALSE) |
| tumorsize | Continuous | Tumor size |
| CO2 | Continuous | CO2 (percents) |
| pain | Continuous | From Gaussian and cut to be integer ranging from 1 to 10 |
| wound | Continuous | From Gaussian and cut to be integer ranging from 1 to 10 |
| mobility | Continuous | From Gaussian and cut to be integer ranging from 1 to 10 |
| ntumors | Continuous | Number of tumors (From Poisson right censored at 9) |
| nmorphine | Continuous | Number of self-administered morphine doses (From Zero-inflated Poisson) |
| lungcapacity | Continuous | Proportion of optimal lung capacity |
| Age | Continuous | In years but recorded at a higher degree of accuracy |
| Married | Categorical | married/living with partner or single |
| FamilyHx | Categorical | Does the patient have a family history of cancer? (yes/no) |
| SmokingHx | Categorical | with three levels, current smoker, former smoker, never smoked |
| Sex | Categorical | female/male |
| CancerStage | Categorical | with four levels, stages 1-4 |
| LengthofStay | Continuous | count number of days patients stayed in the hospital after surgery |
| WBC | Continuous | white blood count |
| RBC | Continuous | red blood count |
| BMI | Continuous | body mass index given by the formula$\frac{KG}{M^{2}}$ |
| IL6 | Continuous | interleukin 6, a proinflammatory cytokine commonly examined as an indicator of inflammation, cannot be lower than zero |
| CRP | Continuous | C-reactive protein, a protein in the blood also used as an indicator of inflammation. It is also impacted by BMI |
| DID | Categorical | Doctor ID |
| Experience | Continuous | years in practice as a doctor |
| School | Categorical | whether the school doctors trained at was high quality or not (Top/Average) |
| Lawsuits | Continuous | Doctors have number of lawsuits |
| HID | Categorical | Hospital ID |
| Medicaid | Continuous | Medicaid at a given hospital |

#### **Table A3: Descriptive statistics for continuous variables**

| **Variable** | N | Mean | STD | Min | Max |
| --- | --- | --- | --- | --- | --- |
| Tumorsize | 8525 | 70.78 | 11.98 | 33.68 | 117.53 |
| Co2 | 8525 | 1.60 | 0.12 | 1.19 | 2.14 |
| Pain | 8525 | 5.52 | 1.48 | 1.00 | 9.00 |
| Wound | 8525 | 5.77 | 1.55 | 1.00 | 9.00 |
| Mobility | 8525 | 5.97 | 1.54 | 1.00 | 9.00 |
| Ntumors | 8525 | 3.04 | 2.53 | 0.00 | 9.00 |
| Nmorphine | 8525 | 3.66 | 2.56 | 0.00 | 15.00 |
| Lungcapacity | 8525 | 0.77 | 0.18 | 0.06 | 1.00 |
| Age | 8525 | 51.09 | 6.37 | 27.40 | 75.91 |
| LengthofStay | 8525 | 5.51 | 1.04 | 2.00 | 9.00 |
| WBC | 8525 | 5992.00 | 997.95 | 2201.00 | 9660.00 |
| RBC | 8525 | 5.00 | 0.28 | 3.85 | 6.02 |
| BMI | 8525 | 29.07 | 6.65 | 18.38 | 58.00 |
| IL6 | 8525 | 4.02 | 2.86 | 0.04 | 23.73 |
| CRP | 8525 | 4.97 | 3.11 | 0.05 | 28.74 |
| Experience | 8525 | 17.64 | 4.08 | 7.00 | 29.00 |
| Lawsuits | 8525 | 1.87 | 1.49 | 0.00 | 9.00 |
| Medicaid | 8525 | 0.51 | 0.21 | 0.14 | 0.82 |

| **Variable** | N | Group | Count | Percent |
| --- | --- | --- | --- | --- |
| remission | 8525 | yes | 2502 | 29.34% |
| Married | 8525 | yes | 5115 | 60.00% |
| FamilyHx | 8525 | yes | 1705 | 20.00% |
| SmokingH$x^{a}$ | 8525 | current | 1705 | 20.00% |
| SmokingH$x^{a}$ | 8525 | never | 5115 | 60.00% |
| Sex | 8525 | male | 3410 | 40.00% |
| CancerStag$e^{b}$ | 8525 | I | 2558 | 30.01% |
| CancerStag$e^{b}$ | 8525 | II | 3409 | 39.99% |
| CancerStag$e^{b}$ | 8525 | III | 1705 | 20.00% |
| School | 8525 | top | 2120 | 24.87% |

#### **Table A4.** **Descriptive statistics for categorical variables**

${}^{a}$The reference group for SmokingHx is ‘former’.

${}^{b}$The reference group for CancerStage is ‘IV’.

#### **Table A5. The coding order of all variables**

| **No** | **Variable** |
| --- | --- |
| 1 | tumorsize |
| 2 | CO2 |
| 3 | pain |
| 4 | wound |
| 5 | mobility |
| 6 | ntumors |
| 7 | nmorphine |
| 8 | lungcapacity |
| 9 | Age |
| 10 | Married |
| 11 | FamilyHx |
| 12 | SmokingHx |
| 13 | Sex |
| 14 | CancerStage |
| 15 | LengthofStay |
| 16 | WBC |
| 17 | RBC |
| 18 | BMI |
| 19 | IL6 |
| 20 | CRP |
| 21 | DID |
| 22 | Experience |
| 23 | School |
| 24 | Lawsuits |
| 25 | HID |
| 26 | Medicaid |

**Figure A1: Flow char of the StepsVM (assuming the number of predictors is “m”)**

Step 1

Fit all $C_{2}^{m}$ SVM models that contain exactly 2 predictors.

Step 2

Choose the highest accuracy among these $C_{2}^{m}$ SVM models.

Step 3

Retain the best two features and then search for the next most significant feature.

Step 4

Choose the highest accuracy among these m-j SVM models.

j = 2, 3, 4, … , m.

Step 5

Repeat this process from Step 3 to 4, until the accuracy drops or the maximum number of predictors are reached.
